# 3-Amino­pyridinium 4-hydr­oxy-3-iodo­naphthalene-1-sulfonate dihydrate

**DOI:** 10.1107/S1600536808014098

**Published:** 2008-05-17

**Authors:** Yun Liu, Jie Li

**Affiliations:** a Basic Experiment Teaching Center, Henan University, Kaifeng 475001, People’s Republic of China

## Abstract

In the hydrated title salt, C_5_H_7_N_2_^+^·C_10_H_6_IO_4_S^−^·2H_2_O, the component species are linked by O—H⋯O and N—H⋯O hydrogen bonds, forming an infinite three-dimensional framework.

## Related literature

For background, see: Li (2007 ▶).
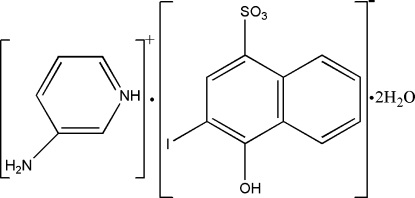


## Experimental

### 

#### Crystal data

C_5_H_7_N_2_^+^·C_10_H_6_IO_4_S^−^·2H_2_O
*M* *_r_* = 480.27
Monoclinic, 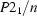

*a* = 15.0219 (6) Å
*b* = 6.9917 (3) Å
*c* = 18.0729 (7) Å
β = 110.868 (1)°
*V* = 1773.66 (12) Å^3^
*Z* = 4
Mo *K*α radiation
μ = 1.96 mm^−1^
*T* = 296 (2) K
0.18 × 0.12 × 0.10 mm

#### Data collection

Bruker SMART APEX CCD diffractometer
Absorption correction: multi-scan (*SADABS*; Bruker, 2001 ▶) *T*_min_ = 0.720, *T*_max_ = 0.828
17926 measured reflections
3484 independent reflections
3128 reflections with *I* > 2σ(*I*)
*R*_int_ = 0.027

#### Refinement

*R*[*F* ^2^ > 2σ(*F* ^2^)] = 0.025
*wR*(*F* ^2^) = 0.069
*S* = 1.08
3484 reflections
258 parameters
34 restraints
H atoms treated by a mixture of independent and constrained refinement
Δρ_max_ = 0.70 e Å^−3^
Δρ_min_ = −0.96 e Å^−3^

### 

Data collection: *SMART* (Bruker, 2001 ▶); cell refinement: *SAINT-Plus* (Bruker, 2001 ▶); data reduction: *SAINT-Plus*; program(s) used to solve structure: *SHELXS97* (Sheldrick, 2008 ▶); program(s) used to refine structure: *SHELXL97* (Sheldrick, 2008 ▶); molecular graphics: *PLATON* (Spek, 2003 ▶); software used to prepare material for publication: *PLATON*.

## Supplementary Material

Crystal structure: contains datablocks global, I. DOI: 10.1107/S1600536808014098/hb2719sup1.cif

Structure factors: contains datablocks I. DOI: 10.1107/S1600536808014098/hb2719Isup2.hkl

Additional supplementary materials:  crystallographic information; 3D view; checkCIF report

## Figures and Tables

**Table 1:** Hydrogen-bond geometry (Å, °)

*D*—H⋯*A*	*D*—H	H⋯*A*	*D*⋯*A*	*D*—H⋯*A*
N1—H1*A*⋯O2*W*	0.882 (10)	1.853 (13)	2.725 (4)	170 (4)
N2—H2*B*⋯O1	0.886 (10)	2.232 (16)	3.085 (3)	162 (4)
N2—H2*C*⋯O2^i^	0.888 (10)	2.179 (11)	3.064 (4)	175 (3)
O4—H4⋯O1*W*	0.843 (10)	1.89 (2)	2.655 (3)	150 (4)
O1*W*—H1*WB*⋯O3^ii^	0.847 (10)	1.983 (12)	2.822 (3)	171 (4)
O1*W*—H1*WA*⋯O4^iii^	0.851 (10)	2.158 (15)	2.944 (3)	153 (3)
O2*W*—H2*WA*⋯O1^iv^	0.853 (10)	1.981 (12)	2.820 (3)	167 (3)
O2*W*—H2*WB*⋯O2^v^	0.856 (10)	1.944 (11)	2.796 (3)	173 (3)

Symmetry codes: (i) 
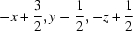
; (ii) 
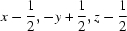
; (iii) 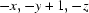; (iv) 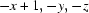; (v) 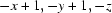.

**Fig. 1.**
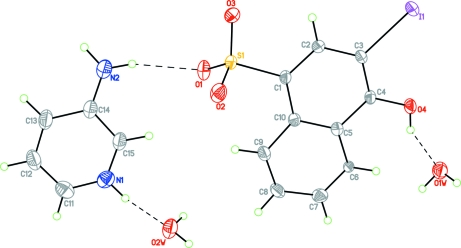
The molecular structure of (I). Displacement ellipsoids for the non-H atoms are drawn at the 50% probability level. Hydrogen bonds are shown as dashed lines.

**Fig. 2.**
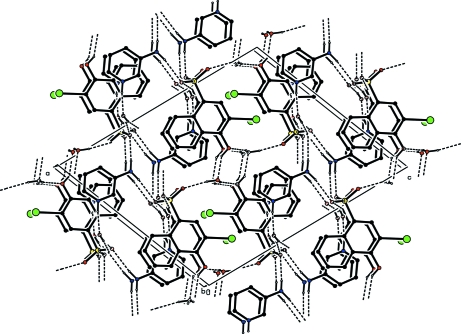
Part of the packing of (I) viewed along the direction [010]. Hydrogen bonds are shown as dashed lines. H atoms not involved in hydrogen bonds have been omitted for clarity.

## supplementary crystallographic information

### Comment

This work continues our previous synthetic and structural studies of the hydrogen bonding interactions between various organic acids and substituted pyridines (Li, 2007).

The asymmetric unit of the title salt, (I), is composed of one 3-aminopyridinium cation, one 8-hydroxy-7-iodo-5-quinolinesulfonate anion and two water molecules (Fig. 1). One water molecule (O1W), acting as a hydrogen bonding donor interacts with the hydroxy oxygen (O4) acting as hydrogen bonding acceptor (Table 1). The other water molecule (O2W) and the 3-aminopyridinium cation are linked by a N1—H1A···O2W hydrogen bond. Moreover, the cation and anion are linked together by a N2—H2B···O1 hydrogen bond. Overall, an infinite three-dimensional framework results (Fig. 2).

### Experimental

A 5-ml ethanol solution of 3-aminopyridine (1.0 mmol, 0.094 g) was added to a 20-ml hot aqueous solution of 8-hydroxy-7-iodo-5-quinolinesulfonic acid (1.0 mmol, 0.351 g) and the mixture was stirred for 20 minutes at 373 K. Then the solution was filtered, and the filtrate was kept at the room temperature. After two weeks, yellow blocks of (I) were obtained.

### Refinement

The N- and O-bonded H atoms bonded were located in a difference synthesis and refined isotropically with N—H = 0.89 (1), O—H = 0.85 (1) and H···H = 1.34 (1) Å, respectively. All the remaining H atoms were placed in calculated positions, with C—H = 0.93Å and were refined as riding with *U*_iso_ = 1.2*U*_eq_(C).   This refinement scheme results in a short intramolecular H4···H6 contact of 1.71Å, although H4 participates in a plausible intermolecular hydrogen bond: thus the location of H4 should be regarded as less certain.  Other placement schemes for H4 appear to lead to disordered hydrogen bond arrangements.

### Crystal data

**Table:**

C_5_H_7_N_2_^+^·C_10_H_6_IO_4_S^–^·2H_2_O	*F*_000_ = 952
*M**_r_* = 480.27	*D*_x_ = 1.799 Mg m^−^^3^
Monoclinic, *P*2_1_/*n*	Mo *K*α radiation λ = 0.71073 Å
Hall symbol: -P 2yn	Cell parameters from 9093 reflections
*a* = 15.0219 (6) Å	θ = 2.4–27.7º
*b* = 6.9917 (3) Å	µ = 1.96 mm^−^^1^
*c* = 18.0729 (7) Å	*T* = 296 (2) K
β = 110.868 (1)º	Block, yellow
*V* = 1773.66 (12) Å^3^	0.18 × 0.12 × 0.10 mm
*Z* = 4	

### Data collection

**Table:**

Bruker SMART APEX CCD diffractometer	3484 independent reflections
Radiation source: fine-focus sealed tube	3128 reflections with *I* > 2σ(*I*)
Monochromator: graphite	*R*_int_ = 0.027
*T* = 296(2) K	θ_max_ = 26.0º
ω scans	θ_min_ = 2.2º
Absorption correction: multi-scan(SADABS; Bruker, 2001)	*h* = −18→18
*T*_min_ = 0.720, *T*_max_ = 0.828	*k* = −8→8
17926 measured reflections	*l* = −22→22

### Refinement

**Table:**

Refinement on *F*^2^	Secondary atom site location: difference Fourier map
Least-squares matrix: full	Hydrogen site location: inferred from neighbouring sites
*R*[*F*^2^ > 2σ(*F*^2^)] = 0.025	H atoms treated by a mixture of independent and constrained refinement
*wR*(*F*^2^) = 0.069	*w* = 1/[σ^2^(*F*_o_^2^) + (0.0364*P*)^2^ + 1.4355*P*] where *P* = (*F*_o_^2^ + 2*F*_c_^2^)/3
*S* = 1.08	(Δ/σ)_max_ < 0.001
3484 reflections	Δρ_max_ = 0.71 e Å^−^^3^
258 parameters	Δρ_min_ = −0.96 e Å^−^^3^
34 restraints	Extinction correction: none
Primary atom site location: structure-invariant direct methods	

### Special details

**Table:**

Geometry. All e.s.d.'s (except the e.s.d. in the dihedral angle between two l.s. planes) are estimated using the full covariance matrix. The cell e.s.d.'s are taken into account individually in the estimation of e.s.d.'s in distances, angles and torsion angles; correlations between e.s.d.'s in cell parameters are only used when they are defined by crystal symmetry. An approximate (isotropic) treatment of cell e.s.d.'s is used for estimating e.s.d.'s involving l.s. planes.
Refinement. Refinement of *F*^2^ against ALL reflections. The weighted *R*-factor *wR* and goodness of fit *S* are based on *F*^2^, conventional *R*-factors *R* are based on *F*, with *F* set to zero for negative *F*^2^. The threshold expression of *F*^2^ > σ(*F*^2^) is used only for calculating *R*-factors(gt) *etc*. and is not relevant to the choice of reflections for refinement. *R*-factors based on *F*^2^ are statistically about twice as large as those based on *F*, and *R*- factors based on ALL data will be even larger.

### Fractional atomic coordinates and isotropic or equivalent isotropic displacement parameters (Å^2^)

**Table:**

	*x*	*y*	*z*	*U*_iso_*/*U*_eq_	
N1	0.7208 (2)	0.1965 (4)	−0.01736 (18)	0.0486 (7)	
H1A	0.6708 (18)	0.233 (5)	−0.0582 (16)	0.056 (11)*	
N2	0.7670 (2)	0.0577 (5)	0.18478 (18)	0.0614 (8)	
H2B	0.7069 (11)	0.034 (6)	0.179 (2)	0.065 (11)*	
H2C	0.8150 (18)	0.008 (5)	0.2246 (15)	0.059 (10)*	
C11	0.8061 (3)	0.1948 (5)	−0.0234 (2)	0.0564 (9)	
H11A	0.8135	0.2277	−0.0708	0.068*	
C12	0.8834 (2)	0.1434 (5)	0.0416 (2)	0.0577 (9)	
H12A	0.9438	0.1405	0.0384	0.069*	
C13	0.8718 (2)	0.0965 (5)	0.1112 (2)	0.0541 (8)	
H13A	0.9245	0.0618	0.1550	0.065*	
C14	0.7811 (2)	0.1006 (4)	0.11680 (19)	0.0433 (7)	
C15	0.7058 (2)	0.1516 (4)	0.0493 (2)	0.0447 (7)	
H15A	0.6443	0.1547	0.0502	0.054*	
S1	0.51967 (4)	0.21097 (9)	0.19020 (4)	0.03081 (15)	
I1	0.159514 (12)	0.20253 (3)	0.230170 (10)	0.03822 (8)	
O1	0.54976 (13)	0.0659 (3)	0.14622 (13)	0.0469 (5)	
O2	0.55842 (13)	0.3982 (3)	0.18257 (13)	0.0469 (5)	
O3	0.53822 (15)	0.1580 (4)	0.27109 (13)	0.0570 (6)	
O4	0.10004 (13)	0.2998 (3)	0.04865 (12)	0.0393 (5)	
H4	0.080 (3)	0.315 (6)	−0.0008 (7)	0.071 (13)*	
C1	0.39401 (17)	0.2327 (4)	0.14385 (15)	0.0273 (5)	
C2	0.33925 (18)	0.2092 (3)	0.18943 (15)	0.0285 (5)	
H2A	0.3682	0.1784	0.2427	0.034*	
C3	0.24002 (18)	0.2308 (4)	0.15712 (15)	0.0281 (5)	
C4	0.19533 (17)	0.2770 (3)	0.07873 (15)	0.0275 (5)	
C5	0.25099 (18)	0.3043 (3)	0.03008 (15)	0.0275 (5)	
C6	0.20225 (16)	0.3533 (3)	−0.04661 (13)	0.0230 (5)	
H6	0.1364	0.3671	−0.0655	0.028*	
C7	0.2508 (2)	0.3803 (4)	−0.09292 (16)	0.0399 (6)	
H7	0.2179	0.4140	−0.1454	0.048*	
C8	0.3502 (2)	0.3611 (5)	−0.06744 (17)	0.0423 (7)	
H8	0.3818	0.3819	−0.1027	0.051*	
C9	0.4003 (2)	0.3120 (4)	0.00897 (17)	0.0371 (6)	
H9	0.4661	0.2985	0.0262	0.045*	
C10	0.35128 (18)	0.2815 (3)	0.06225 (15)	0.0275 (5)	
O1W	−0.02157 (17)	0.3572 (4)	−0.09765 (13)	0.0532 (6)	
H1WA	−0.038 (2)	0.473 (2)	−0.095 (2)	0.060 (5)*	
H1WB	0.002 (2)	0.359 (4)	−0.1337 (16)	0.056 (5)*	
O2W	0.55451 (16)	0.2736 (3)	−0.13855 (17)	0.0555 (6)	
H2WA	0.5162 (18)	0.179 (3)	−0.147 (2)	0.059 (11)*	
H2WB	0.5167 (18)	0.370 (3)	−0.151 (2)	0.072 (13)*	

### Atomic displacement parameters (Å^2^)

**Table:**

	*U*^11^	*U*^22^	*U*^33^	*U*^12^	*U*^13^	*U*^23^
N1	0.0482 (16)	0.0385 (14)	0.0518 (16)	−0.0003 (12)	0.0091 (13)	0.0011 (12)
N2	0.0386 (16)	0.090 (2)	0.0548 (17)	0.0127 (16)	0.0150 (14)	0.0124 (16)
C11	0.063 (2)	0.0483 (19)	0.064 (2)	−0.0091 (16)	0.0302 (19)	0.0008 (16)
C12	0.0395 (17)	0.060 (2)	0.080 (3)	−0.0068 (15)	0.0282 (18)	0.0007 (19)
C13	0.0307 (15)	0.058 (2)	0.068 (2)	0.0012 (14)	0.0112 (15)	0.0044 (17)
C14	0.0321 (14)	0.0431 (16)	0.0531 (17)	0.0027 (12)	0.0129 (13)	−0.0010 (14)
C15	0.0316 (14)	0.0430 (16)	0.0580 (19)	0.0040 (12)	0.0142 (14)	0.0016 (14)
S1	0.0198 (3)	0.0380 (3)	0.0329 (3)	0.0003 (2)	0.0073 (2)	0.0048 (3)
I1	0.03151 (12)	0.05175 (14)	0.03694 (12)	0.00166 (7)	0.01898 (8)	0.00622 (8)
O1	0.0299 (10)	0.0489 (12)	0.0630 (13)	0.0051 (9)	0.0180 (9)	−0.0058 (10)
O2	0.0289 (10)	0.0424 (11)	0.0616 (13)	−0.0083 (9)	0.0066 (9)	0.0054 (10)
O3	0.0303 (11)	0.0986 (19)	0.0378 (11)	0.0063 (11)	0.0069 (9)	0.0226 (12)
O4	0.0214 (9)	0.0645 (14)	0.0313 (11)	0.0048 (8)	0.0084 (8)	0.0037 (9)
C1	0.0206 (11)	0.0297 (12)	0.0318 (13)	0.0004 (9)	0.0095 (10)	0.0015 (10)
C2	0.0258 (12)	0.0323 (13)	0.0261 (12)	0.0011 (10)	0.0078 (10)	0.0036 (10)
C3	0.0249 (12)	0.0336 (13)	0.0297 (12)	−0.0003 (10)	0.0144 (10)	0.0007 (10)
C4	0.0214 (12)	0.0318 (12)	0.0297 (12)	0.0013 (10)	0.0095 (10)	−0.0014 (10)
C5	0.0255 (12)	0.0278 (12)	0.0296 (12)	−0.0006 (9)	0.0101 (10)	−0.0019 (9)
C6	0.0162 (10)	0.0326 (12)	0.0178 (10)	0.0005 (9)	0.0030 (8)	0.0016 (9)
C7	0.0390 (15)	0.0479 (16)	0.0289 (13)	−0.0033 (13)	0.0075 (11)	0.0030 (12)
C8	0.0375 (15)	0.0600 (18)	0.0342 (14)	−0.0037 (14)	0.0188 (12)	0.0049 (13)
C9	0.0274 (13)	0.0495 (16)	0.0362 (14)	−0.0016 (12)	0.0135 (12)	0.0028 (12)
C10	0.0248 (12)	0.0285 (12)	0.0288 (12)	−0.0015 (9)	0.0092 (10)	−0.0006 (10)
O1W	0.0462 (12)	0.0741 (16)	0.0393 (12)	0.0129 (12)	0.0151 (10)	0.0012 (11)
O2W	0.0377 (12)	0.0424 (13)	0.0792 (18)	0.0006 (10)	0.0122 (12)	−0.0025 (12)

### Geometric parameters (Å, °)

**Table:**

N1—C11	1.325 (5)	C1—C2	1.366 (4)
N1—C15	1.339 (4)	C1—C10	1.424 (4)
N1—H1A	0.882 (10)	C2—C3	1.402 (4)
N2—C14	1.353 (4)	C2—H2A	0.9300
N2—H2B	0.886 (10)	C3—C4	1.373 (4)
N2—H2C	0.888 (10)	C4—C5	1.425 (4)
C11—C12	1.374 (5)	C5—C6	1.361 (3)
C11—H11A	0.9300	C5—C10	1.417 (4)
C12—C13	1.369 (5)	C6—C7	1.305 (4)
C12—H12A	0.9300	C6—H6	0.9300
C13—C14	1.403 (4)	C7—C8	1.404 (4)
C13—H13A	0.9300	C7—H7	0.9300
C14—C15	1.382 (4)	C8—C9	1.360 (4)
C15—H15A	0.9300	C8—H8	0.9300
S1—O3	1.436 (2)	C9—C10	1.421 (4)
S1—O1	1.456 (2)	C9—H9	0.9300
S1—O2	1.459 (2)	O1W—H1WA	0.851 (10)
S1—C1	1.778 (2)	O1W—H1WB	0.847 (10)
I1—C3	2.093 (2)	O2W—H2WA	0.853 (10)
O4—C4	1.347 (3)	O2W—H2WB	0.856 (10)
O4—H4	0.843 (10)		
			
C11—N1—C15	123.4 (3)	C10—C1—S1	121.29 (19)
C11—N1—H1A	120 (3)	C1—C2—C3	121.0 (2)
C15—N1—H1A	117 (3)	C1—C2—H2A	119.5
C14—N2—H2B	115 (2)	C3—C2—H2A	119.5
C14—N2—H2C	119 (2)	C4—C3—C2	120.8 (2)
H2B—N2—H2C	122 (4)	C4—C3—I1	119.55 (18)
N1—C11—C12	118.6 (4)	C2—C3—I1	119.64 (19)
N1—C11—H11A	120.7	O4—C4—C3	120.2 (2)
C12—C11—H11A	120.7	O4—C4—C5	120.5 (2)
C13—C12—C11	120.3 (3)	C3—C4—C5	119.3 (2)
C13—C12—H12A	119.9	C6—C5—C10	123.6 (2)
C11—C12—H12A	119.9	C6—C5—C4	116.2 (2)
C12—C13—C14	120.4 (3)	C10—C5—C4	120.2 (2)
C12—C13—H13A	119.8	C7—C6—C5	118.0 (2)
C14—C13—H13A	119.8	C7—C6—H6	121.0
N2—C14—C15	121.0 (3)	C5—C6—H6	121.0
N2—C14—C13	122.1 (3)	C6—C7—C8	123.2 (3)
C15—C14—C13	116.9 (3)	C6—C7—H7	118.4
N1—C15—C14	120.5 (3)	C8—C7—H7	118.4
N1—C15—H15A	119.8	C9—C8—C7	119.7 (3)
C14—C15—H15A	119.8	C9—C8—H8	120.1
O3—S1—O1	113.00 (15)	C7—C8—H8	120.1
O3—S1—O2	112.85 (15)	C8—C9—C10	119.6 (3)
O1—S1—O2	111.19 (13)	C8—C9—H9	120.2
O3—S1—C1	106.86 (12)	C10—C9—H9	120.2
O1—S1—C1	106.64 (12)	C5—C10—C9	115.9 (2)
O2—S1—C1	105.73 (12)	C5—C10—C1	118.2 (2)
C4—O4—H4	111 (3)	C9—C10—C1	125.9 (2)
C2—C1—C10	120.5 (2)	H1WA—O1W—H1WB	103.8 (15)
C2—C1—S1	118.17 (19)	H2WA—O2W—H2WB	102.6 (15)

### Hydrogen-bond geometry (Å, °)

**Table:**

*D*—H···*A*	*D*—H	H···*A*	*D*···*A*	*D*—H···*A*
N1—H1A···O2W	0.882 (10)	1.853 (13)	2.725 (4)	170 (4)
N2—H2B···O1	0.886 (10)	2.232 (16)	3.085 (3)	162 (4)
N2—H2C···O2^i^	0.888 (10)	2.179 (11)	3.064 (4)	175 (3)
O4—H4···O1W	0.843 (10)	1.89 (2)	2.655 (3)	150 (4)
O1W—H1WB···O3^ii^	0.847 (10)	1.983 (12)	2.822 (3)	171 (4)
O1W—H1WA···O4^iii^	0.851 (10)	2.158 (15)	2.944 (3)	153 (3)
O2W—H2WA···O1^iv^	0.853 (10)	1.981 (12)	2.820 (3)	167 (3)
O2W—H2WB···O2^v^	0.856 (10)	1.944 (11)	2.796 (3)	173 (3)

Symmetry codes: (i) −*x*+3/2, *y*−1/2, −*z*+1/2; (ii) *x*−1/2, −*y*+1/2, *z*−1/2; (iii) −*x*, −*y*+1, −*z*; (iv) −*x*+1, −*y*, −*z*; (v) −*x*+1, −*y*+1, −*z*.
